# Social support, anxiety symptoms, and depression symptoms among residents in standardized residency training programs: the mediating effects of emotional exhaustion

**DOI:** 10.1186/s12888-021-03381-1

**Published:** 2021-09-21

**Authors:** Hui Zhang, Nianqi Cui, Dandan Chen, Ping Zou, Jing Shao, Xiyi Wang, Yichi Zhang, Jiao Du, Chunxue Du, Deyi Zheng

**Affiliations:** 1 Department of cardiology, Guizhou Provincial People’s Hospital, Guiyang, China; 2 The Second Affiliated Hospital Zhejiang University School of Medicine, Hangzhou, China; 3 Zhejiang university School of Medicine Sir Run Run Shaw Hospital, Hangzhou, China; 4 School of Nursing, Nipissing University, 222 St. Patrick Street, Toronto, Ontario Canada; 5 School of Nursing, Shanghai JiaoTong University, Shanghai, China; 6 Department of Burn and Plastic Surgery, Guizhou Provincial People’s Hospital, Guiyang, China

**Keywords:** Anxiety symptoms, Depression symptoms,, Emotional exhaustion, Social support, Residents

## Abstract

**Background:**

Although studies indicate that social support is related to emotional exhaustion, depression symptoms, and anxiety symptoms, the underlying mechanism between those variables remains unknown.

**Methods:**

Based on a sample of 254 residents in standardized residency training programs, two mediation models were tested in which emotional exhaustion served as a mediator in the relationship between social support and anxiety symptoms/depression symptoms. We used the following self-reported questionnaires as instruments to collect data: zung self-rating depression scale, zung self-rating anxiety scale, social support rating scale, and emotional exhaustion scale.

**Results:**

In the final study sample, the mean age of the residents was 25.92 years old (SD =1.88), and a total of 41.3% were male, and 58.7% were female. This current study suggested that social support was proven to be a relevant factor affecting anxiety symptoms and depression symptoms. Particularly, the results also indicated that emotional exhaustion partially mediated the impact of social support on anxiety symptoms and depression symptoms among Chinese residents in the standardized residency training program.

**Conclusions:**

Our study signifies that enhancements in social support and reduction of emotional exhaustion can directly or indirectly affect anxiety symptoms and depression symptoms among Chinese residents in the standardized residency training program. These findings will offer insight for health-sector managers to develop programs aimed at social support and adopt individual-level interventions and organization-level interventions to reduce emotional exhaustion.

## Introduction

Over the last decade, the National Health and Family Planning Commission have been progressively promoting the standardized residency training program (Standardized Residency Training Program, SRTP) to improve the quality of physician training in China [1]. All new medical graduates looking for jobs in hospitals are required to complete the SRTP. In the SRTP, residents are expected to master learning skills, patient care skills, teaching skills, and documentation skills, despite long working hours and demanding needs of patients [2]. Those residents are more likely to experience health problems due to a high-pressure job [1].

The most common strain for residents is burnout. According to a meta-analysis published in 2019, the global prevalence of burnout among medical residents is 50.13% [2]. Research has suggested that emotional exhaustion is the core dimension of burnout, and this means that emotional exhaustion is more closely related to outcomes than other dimensions of burnout [3, 4]. Emotional exhaustion is defined as the feeling of being emotionally exhausted by one’s work, and individuals with emotional exhaustion will have physical fatigue and a sense of feeling emotionally “drained”. Neglected emotional exhaustion can cause anxiety, depression, discontinuation of residency, fatigue, and insomnia [5]. To deliver high-quality healthcare, it is important to pay attention to emotional exhaustion among residents in the SRTP.

Social support refers to “those social interactions or relationships that provide individuals with actual assistance or that embed individuals within a social system believed to provide love, care, or sense of attachment to a valued social group or dyad” [6]. Social support consists of received social support and perceived social support [7]. Received support is when an individual is obtaining actual help from others whereas perceived support is the belief that helping behaviors will be available when individuals are in need [7]. Studies suggested that social support can protect against stress and improve mental health [8, 9]. For instance, Li et al. indicated that social support was negatively associated with mental health [10]. Additionally, from the organization’s perspective, the lack of resources could be the antecedent of emotional exhaustion [11]. In the literature, many empirical studies indicated that social support can be regarded as a helpful resource to deal with demands at work [12]. It is the organization’s obligation to offer employees social support and encourage employees to seek social support from their network by offering programs to cope with work pressure and a heavy workload, as little or no social support to residents in the SRTP might be an important source of emotional exhaustion.

Research also indicated that emotional exhaustion was positively related to anxiety or depression [13]. A large and growing body of literature suggested that physicians, being one of the most stressful and demanding occupations, are more likely to suffer mental health disorders (e.g., anxiety symptoms and depression symptoms) [14]. Especially, residents in the SRTP could be most vulnerable to anxiety and depression, because they may not be capable of heavy workloads, the morbidity and mortality of patients, pressure of patient and service demands, and challenging daily work routines [15]. A previous study has shown that the rate of depression was 28.3% among residents in the SRTP [16]. Given that mental health can have a detrimental impact on residents in the SRTP and health services, including quality of patient care and medical errors, research needs to identify the mechanism between social support, emotional exhaustion, and depression/anxiety to inform effective interventions.

Although studies indicated that social support was related to emotional exhaustion, and emotional exhaustion was associated with depression symptoms and anxiety symptoms, the underlying mechanism between those variables remains unknown. This study aimed to focus on emotional exhaustion as one mechanism that explains the relationships between social support and anxiety and depression symptoms. In the context of the SRTP implementation, the results of this study contribute to a better understanding of the underlying mechanism between these important variables for residents in the SRTP. Ultimately, the well-being of residents in the SRTP can be improved in order to offer safe patient care and maintain a high-quality healthcare system.

### Theoretical background

According to conservation of resources (COR) theory, “individuals strive to obtain, retain, foster, and protect those things they centrally value” [17]. Central value includes self-preservation, a positive sense of self, well-being, and so on. This means that people promote their well-being by employing key resources. If key resources are not sufficient and this process is failed, their well-being cannot be ultimately protected. On the basis of COR theory, strain can occur when key resources are lacking [17]. Empirical research has shown that low levels of social support are associated with a high level of emotional exhaustion [18]. In other words, an important resource such as the lack of social support is responsible for the development of emotional exhaustion. Furthermore, individuals with fewer resources are more vulnerable to resource loss and the detrimental psychological effect of losing resources can lead to mental health issues [19]. Emotional exhaustion which is conceptualized as representing a deficit in resources was positively associated with mental health [20]. Moreover, the mediating role of emotional exhaustion in the relationships between resources and mental health has been proven. For example, Huang et al. found that job control regarded as one of the resources had indirect effects on mental health through emotional exhaustion [3]. Thus, we predicted the following:

• Hypothesis 1: Emotional exhaustion mediates the negative relationship between social support and anxiety symptoms.

• Hypothesis 2: Emotional exhaustion mediates the negative relationship between social support and depression symptoms.

## Methods

### Study units and participants

The current study was conducted among residents in the SRTP in the Guizhou Provincial People’s Hospital, and a convenience sample was used in May 2019. Three trained researchers were responsible for data collection. The researchers contacted managers in the wards to acquire consent to participate in this study. If managers agreed with participation, they were asked to provide the number of residents in the SRTP who were working in their wards. The researchers explained the aim of this research to each resident and residents were informed that the collected data will be kept confidential. Residents were also informed that they had the right to refuse participation. Residents were asked to complete anonymous self-administered paper-pencil questionnaires.

### Measures

#### Anxiety symptoms

The Chinese version of the Zung Self-Rating Anxiety Scale is a valid questionnaire to access anxiety symptoms among the Chinese population [21]. This tool includes 20 items (e.g., “I feel afraid for no reason at all”), and items are scored on a 4-point Likert scale (1 to 4). Higher standard scores reflect high levels of anxiety. The Cronbach’s alpha was 0.855.

#### Depression symptoms

The Chinese version of the Zung Self-Rating Depression Scale was adopted to access depression symptoms [22]. These are 20 items (e.g., “I have trouble sleeping at night”) and those items are scored on a 4-point Likert scale (1 to 4). A higher standard score indicates severe depression symptoms. The Cronbach’s alpha was 0.822.

#### Social support

The 10-item Social Support Rating Scale questionnaire was applied to evaluate social support in our study [23]. This Chinese questionnaire includes three dimensions: (1) objective support, reflecting the support an individual receives in an emergency (e.g. “if risk situations have been identified, you can receive financial, material, or emotional support from your family members, your close friends, or your colleagues”), (2) subjective support, reflecting an individual’s perceived network of friends, neighbors, colleagues, and family members (e.g. “How many close friends do you have?”), and (3) the usage of support, referring to the pattern of behavior that an individual utilizes when seeking social support (e.g. “Do you participate in formal or informal activities?”). Participants are scored on a 4-point scale where the scores range from 12 to 66. Higher scores mean more social support is provided. The Cronbach’s alpha for SSRS was 0.729.

#### Emotional exhaustion

The emotional exhaustion subscale from the Chinese Burnout Inventory was used to measure burnout [24]. This subscale includes 5 items (e.g., “I feel emotionally drained from my work”), and increased scores suggest a high level of emotional exhaustion. Participants responded to five items on this scale ranging from 0 to 6. The Cronbach’s alpha was 0.946.

### Statistical analysis

SPSS software (version 24.0) was used for data analysis, we calculated means, standard deviations (SD), the Cronbach’s alpha, average variance extracted (AVE), and correlation matrix. PROCESS macro (v3.3) for SPSS was used to perform mediation analyses, and model 4 of PROCESS macro was appropriate for the mediation model. In a mediation model [25], X (an independent variable) is related to Y(a dependent variable) when accounting for M (a mediator), and this path coefficient denoted *c’* is the direct effect. X is related to M (a mediator), and this path coefficient is denoted *a*. M is related to Y, and this path coefficient is denoted *b*. The path coefficient for *ab* is indirect effects, and the indirect effect is significant when zero is not included within the 95% bias-corrected confidence intervals [25]. The significance of the indirect effect was based 5000 bootstrapping resample s[26]. Demographic variables (e.g., age, gender, education, years of SRTP, and marital status) were controlled in the mediation analysis for dependent variables.

## Results

A total of 310 residents were recruited, and thirty residents refused participation. Twenty-six residents failing to provide information about the key variables examined were excluded. Consequently, 254 residents were included in the final study sample.

The mean age of the residents was 25.92 years old (SD =1.88), and a total of 41.3% were male, and 58.7% were female. In this study, 36.6% were in the first year of SRTP, and 37% were in the second year of SRTP, and 26.4% were in the third year of SRTP. 15.7% were married, and 84.3% reported they were single. The sample included 51 (20.1%) participants with postgraduate degrees and 203 (79.9%) participants with bachelor’s degrees. Table 1 presents means, standard deviations (SD), the Cronbach’s alpha, AVE, and correlation matrix for all important variables. Because all AVE ranged from 0.50 and above, convergent validity was satisfactory for constructs. The discriminant validity was satisfactory, as the square root of AVE values for social support, emotional exhaustion, anxiety symptoms, and depression symptoms exceeded the construct correlation values.

**Table 1:** Correlation coefficient, mean, standard deviation, and AVE (*N* = 254)

Variables	M	SD	The Cronbach’s a	AVE	1	2	3	4
1 SS	2.20	0.53	0.73	0.53	**0.73**			
2 EE	2.50	1.55	0.95	0.86	−0.33**	**0.93**		.
3 Anxiety	1.87	0.53	0.86	0.62	−0.30**	0.49**	**0.79**	
4Depression	2.15	0.57	0.82	0.56	−0.28**	0.49**	0.72**	**0.75**

*SS* social support, *EE* emotional exhaustion, **Significant at the 0.01 level; the square of root of AVE values are bolded, *AVE* average variance extracted

### Mediation analyses

**Figure 1:**
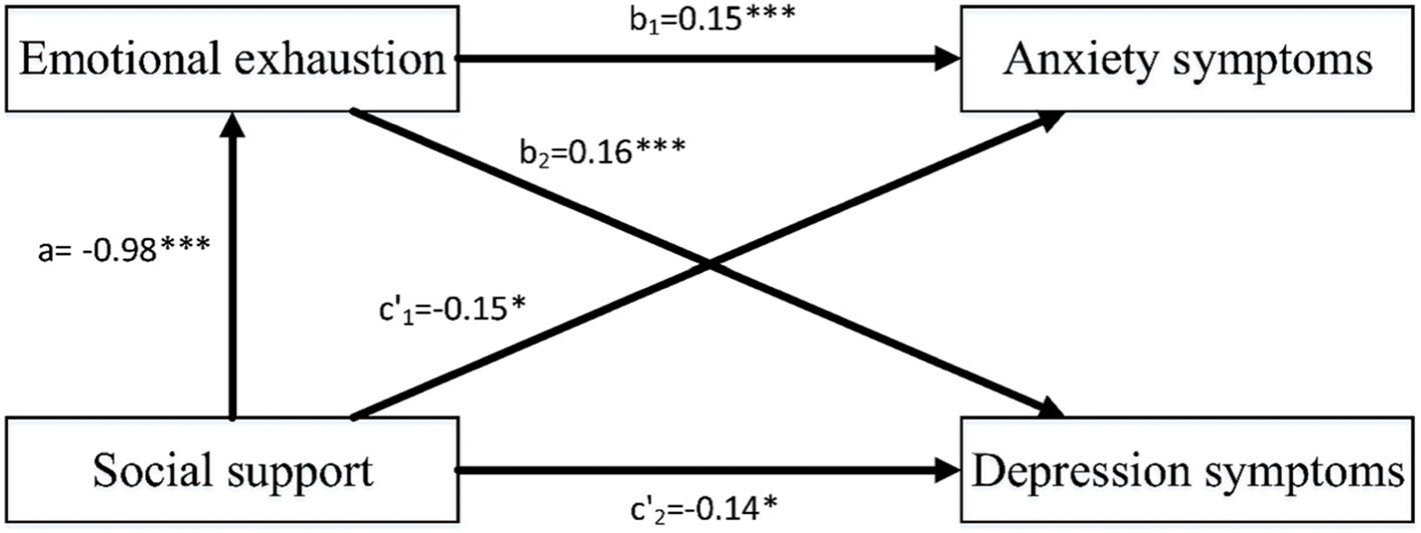
The mediation model. a = direct effect of social support on emotional exhaustion; b_1_ = direct effect of emotional exhaustion on anxiety symptoms; b_2_ = direct effect of emotional exhaustion on depression; c′_1_ = direct effect of social support on anxiety when accounting for emotional exhaustion; c′_2_ = direct effect of social support on depression direct effect of social support on depression symptoms when accounting for emotional exhaustion. Age, gender, education, years of SRTP, and marital status were controlled in the mediation analysis for dependent variables; *p < 0.05, ***p < 0.001.

The results of mediation analysis were shown in Table 2 and Fig. 1. Social support was significantly related to anxiety symptoms (*c’*_1_ = − 0.15, SE = 0.06, 95% CI = [− 0.27, − 0.03]) and depression symptoms (*c’*_2=_ − 0.14, SE = 0.07, 95% CI = [− 0.27, − 0.01]). By using the bias-corrected bootstrap method, the results suggested that after controlling sociodemographic variables, social support affected anxiety symptoms through emotional exhaustion significantly, *ab*_*1*_ = − 0.14, SE = 0.03, 95% CI = [− 0.21, − 0.08]. Hypothesis 1 was supported. Moreover, after controlling sociodemographic variables, social support affected depression symptoms through emotional exhaustion significantly, *ab*_*2*_ = − 0.16, SE = 0.04, 95% CI = [− 0.24, − 0.09]. Hypothesis 2 was supported.

**Table 2:** Mediation analyses

DV	IV	coeff	se	t	LLCI	ULCI	*F*	*R*^*2*^
EE	constant	4.65	0.40	11.68***	3.86	5.43	30.75***	0.11
	SS	−0.98	0.18	−5.55***	−1.32	− 0.63		
Anxiety	constant	3.71	0.75	4.94***	2.23	5.19	14.59***	0.29
	EE	0.15	0.02	7.40***	0.11	0.18		
	SS	−0.15	0.06	−2.45*	−0.27	− 0.03		
	Gender	−0.16	0.06	−2.64**	−0.28	− 0.04		
	Age	−0.05	0.02	−2.42*	− 0.09	− 0.01		
	Years of SRTP	−0.07	0.04	−1.79	−0.15	0.01		
	Marital status	−0.06	0.09	−0.61	− 0.23	0.12		
	Education	−0.07	0.08	−0.88	− 0.22	0.08		
Anxiety	constant	4.91	0.81	6.07***	3.32	6.51	6.48***	0.14
	SS	−0.29	0.06	−4.62***	− 0.42	− 0.17		
	Gender	−0.19	0.07	−2.85**	− 0.32	− 0.06		
	Age	−0.06	0.02	−2.77**	−0.10	− 0.02		
	Years of SRTP	− 0.08	0.05	−1.84	−0.17	0.01		
	Marital status	−0.08	0.10	−0.81	− 0.28	0.12		
	Education	−0.11	0.09	−1.33	−0.28	0.06		
	Indirect effect 1	−**0.14**	**0.03**	–	**−0.21**	**− 0.08**		
EE	constant	4.65	0.40	11.68***	3.86	5.43	30.75***	0.11
	SS	−0.98	0.18	−5.55***	−1.32	−0.63		
Depression	constant	3.44	0.82	4.20***	1.83	5.06	12.99***	0.27
	EE	0.16	0.02	7.54***	0.12	0.20		
	SS	− 0.14	0.07	−2.18*	−0.27	− 0.01		
	Gender	−0.07	0.07	−0.98	− 0.20	0.07		
	Age	−0.04	0.02	−1.85	− 0.08	0.00		
	Years of SRTP	−0.05	0.04	−1.03	− 0.13	0.04		
	Marital status	−0.04	0.10	− 0.37	− 0.23	0.16		
	Education	−0.04	0.09	−0.44	− 0.21	0.13		
Depression	constant	4.78	0.89	5.39***	3.03	6.53	4.63***	0.10
	SS	−0.30	0.07	−4.39***	− 0.44	− 0.17		
	Gender	−0.10	0.07	−1.35	− 0.25	0.05		
	Age	−0.05	0.02	−2.26*	−0.10	− 0.01		
	Years of SRTP	−0.06	0.05	−1.14	−0.15	0.04		
	Marital status	−0.06	0.11	−0.59	− 0.28	0.15		
	Education	−0.09	0.09	−0.94	− 0.27	0.10		
	Indirect effect 2	**−0.16**	**0.04**	–	**− 0.24**	**− 0.09**		

Bootstrap sample size = 5000. *DV* Dependent variable, *IV* Independent variable, *SS* social support, *EE* emotional exhaustion, *ULCI* Upper Limit of Confidence Interval, *LLCI* Lower Limit of Confidence; *p < 0.05, **p < 0.01, ***p < 0.001; Indirect effect1: social support→emotional exhaustion→anxiety symptoms; Indirect effect 2: social support→emotional exhaustion→depression symptoms; The significance of the indirect effect was based 5000 bootstrapping resamples

## Discussion

Resident training can be crucial for medical graduates to develop skills and acquire knowledge before becoming professionals. Greater work pressure and low income will result in mental issuedirect effect of social support on anxietydirect effect of social support on anxietys among thidirect effect of social support on anxietys population. This research aimed to examine if emotional exhaustion mediated the relationships between the negative relationship between social support and anxiety and depression symptoms among Chinese residents in the SRTP. Our results suggested that low levels of social support related to higher levels of anxiety and depression symptoms. Moreover, emotional exhaustion can be seen as a potential mediator of the relationships.

In the current study, social support is an important resource negatively associated with anxiety and depression symptoms among residents of the SRTP. The results were consistent with previous studies. For example, Li et al. found that social support was correlated with mental health [10]. According to COR theory, when the important resource (e.g., social support) is decreased, individuals cannot protect their well-being since losing resources have harmful effects on the psychological state of individuals. Moreover, small amounts of social support limit the individuals’ ability to deal with stressors and other potential life crises, which may also be damaging to individuals’ health. People with sufficient social support will view themselves as valued and deserving of love and appreciation, and social support helps them withstand pressures and seek help [27]. Consequently, they will be more capable to cope with life crises and avoid mental health problems.

The results also indicated that emotional exhaustion can be regarded as a mediator in the relationships between social support and anxiety and depression symptoms. Emotional exhaustion emerges as an underlying mechanism explaining associations between social support and anxiety and depression symptoms. These findings are supported by earlier research examining the mediating effects of emotional exhaustion on the relationships between resources and mental health. A previous study explained the mediating role of emotional exhaustion in the association between job control and mental health [3]. Similarly, Zhang et al. found that the effect of work-family conflict on anxiety symptoms can be mediated by emotional exhaustion [21]. In line with COR theory, Hobfoll and Lilly found that the loss of resources was more closely associated with emotional exhaustion than resource gain [28]. The loss of social support can cause stress and strain, therefore emotional exhaustion can be viewed as one form of strain [17]. In such a case, individuals with emotional exhaustion are more likely to experience further resource losses and are less capable to cope with challenges resulting in a negative long term impact on mental health .

### Practical implications

Our findings have important implications. As social support can directly affect anxiety and depression symptoms, social support interventions should be considered. Different types of social support (instrumental aid, emotional concern, provision of information), may originate from various sources such as supervisors, coworkers, or family members and friends. Individuals are encouraged to cast a wider social network since different relationships for different kinds of support can help people cope with a variety of difficulties. Individuals should also make an effort to get involved in many activities such as groups, clubs, and classes to build their social network. More importantly, people should seek help proactively when they are in need. For residents of the SRTP, it is beneficial for them to seek emotional support from family members and friends, and also seek advice from senior physicians and peers to manage difficult tasks and potential crises.

Due to the mediating role of emotional exhaustion in the relationship between social support and anxiety and depression symptoms, policymakers and hospital management should develop an appropriate intervention for the proper management of emotional exhaustion among residents of the SRTP. From the individual perspective, it is suggested that relaxation training, cognitive behavioral therapy, and mindfulness are helpful to mitigate emotional strain [29]. More importantly, from an organizational perspective, interventions related to workplace risk factors can be more acceptable to participants as a form of universal prevention. The hospital management system should offer early prevention programs to decrease emotional exhaustion among residents in the SRTP. The reduction of job demands can be a core element in such an intervention. This means that the hospital management system should set limits on working hours, reduce workloads, and modify local working conditions, which can in turn lead to effective reductions in emotional strain. By doing this, boundaries between work time and nonwork time can be set, so work-life balance can enable individuals to recover from work. This may be more effective in reducing emotional exhaustion [30]. Additionally, a past study reported that Chinese residents work far more than 40 h a week in hospitals, while their income is pitifully leading to reduced professional dignity and sense of honor, and increased emotional strain [31]. It is imperative for policymakers in China to improve residents’ income to reduce emotional exhaustion.

### Study limitations

There were some limitations in our study. First, this cross-sectional study cannot conclude causal inferences and directionality, and future research is recommended to adopt a longitudinal design for valid conclusions. Second, we only considered social support as an important resource based on the COR theory, therefore future studies should explore other important resources to expand the COR theory. Lastly, the sample size was small in this study and all participants were recruited from a single hospital, which may lead to cause single source bias. Multicenter studies and large sample size are needed in the future.

## Conclusion

This current study underlining the importance of social support, suggests that social support is proven to be a relevant factor affecting anxiety and depression symptoms. Particularly, the results also indicate that emotional exhaustion partially mediates the impact of social support on anxiety and depression symptoms among Chinese residents in the SRTP. Our study signifies that enhancements in social support and reduction of emotional exhaustion can directly or indirectly affect anxiety and depression symptoms among Chinese residents in the SRTP. These findings will offer some insight for health-sector managers to develop programs aimed at improving social support and emotional exhaustion.

## Data Availability

The datasets used and analyzed during the current study are available from the corresponding author on reasonable request.

## Abbreviations

SRTP: Standardized Residency Training Program
COR: Conservation of Resources
DV: Dependent Variable
IV: Independent Variable
SS: Social Support
EE: Emotional Exhaustion
AVE: Average Variance EExtracted
ULCI: Upper Limit of Confidence Interval
LLCI: Lower Limit of Confidence Interval
SPSS: Statistical Package for Social Sciences
